# Cancer cell membrane-wrapped nanoparticles for cancer immunotherapy: A review of current developments

**DOI:** 10.3389/fimmu.2022.973601

**Published:** 2022-08-29

**Authors:** Qi Jiang, Mixue Xie, Ruyin Chen, Feifei Yan, Chanqi Ye, Qiong Li, Shuaishuai Xu, Wei Wu, Yunlu Jia, Peng Shen, Jian Ruan

**Affiliations:** ^1^ Department of Medical Oncology, The First Affiliated Hospital, Zhejiang University School of Medicine, and Key Laboratory of Cancer Prevention and Intervention, Ministry of Education, Hangzhou, China; ^2^ Department of Hematology, The First Affiliated Hospital, Zhejiang University School of Medicine, Hangzhou, China

**Keywords:** cancer cell membrane, membrane-wrapped, nanoparticle, drug delivery, immunotherapy, nanovaccine

## Abstract

**Background:**

As the forefront of nanomedicine, bionic nanotechnology has been widely used for drug delivery in order to obtain better efficacy but less toxicity for cancer treatments. With the rise of immunotherapy, the combination of nanotechnology and immunotherapy will play a greater potential of anti-tumor therapy. Due to its advantage of homologous targeting and antigen library from source cells, cancer cell membrane (CCM)-wrapped nanoparticles (CCNPs) has become an emerging topic in the field of immunotherapy.

**Key scientific concepts of review:**

CCNP strategies include targeting or modulating the tumor immune microenvironment and combination therapy with immune checkpoint inhibitors and cancer vaccines. This review summarizes the current developments in CCNPs for cancer immunotherapy and provides insight into the challenges of transferring this technology from the laboratory to the clinic as well as the potential future of this technology.

**Conclusion:**

This review described CCNPs have enormous potential in cancer immunotherapy, but there are still challenges in terms of translating their effects *in vitro* to the clinical setting. We believe that these challenges can be addressed in the future with a focus on individualized treatment with CCNPs as well as CCNPs combined with other effective treatments.

## Introduction to cancer immunotherapy and cancer cell membrane-wrapped nanoparticles

Cancer immunotherapy is known as the third technological revolution in cancer treatment. Compared with traditional anti-tumor therapies such as surgery, radiotherapy, and chemotherapy, immunotherapy is an innovative treatment that focuses on the use of the body’s own immune mechanism and has shown breakthrough efficacy for patients with recurrent and refractory malignant tumors (1). First of all, cellular immunotherapy, especially chimeric antigen receptor T-cell immunotherapy (CAR-T), is a representative breakthrough in cancer immunotherapy. CAR-T has accomplished remarkable results in the treatment of hematologic tumors, especially in patients with B-cell acute lymphoblastic leukemia, with an initial response rate of more than 90% (2). Hematologic tumors, however, are only a small part of a larger cancer population, with solid tumors accounting for about 90% of cancer cases, according to the latest cancer data published in 2022 (3). Despite the larger prevalence of solid tumors, the efficacy for solid tumors is not as expected due to the heterogeneity within solid tumors and tumor microenvironment (TME). No clinical application of CAR-T for the treatment of solid tumors has been approved by the US FDA. Targeting transforming growth factor (TGF)-β and oncolytic viruses to TME have been evaluated as therapeutic tools to increase the efficacy of CAR-T in metastatic castration-resistant prostate cancer (mCRPC) and solid tumor mouse models of melanoma and glioma (4, 5). Immune checkpoint inhibitors (ICIs) such as programmed death-ligand 1/programmed death-1 (PD-L1/PD-1) blockade and cytotoxic T-lymphocyte-associated protein 4 (CTLA-4) inhibitors are another representative breakthrough in cancer immunotherapy that has been successfully used in solid tumors (6). Ideal cancer vaccines could overcome the immune suppression in tumors and induce both humoral immunity and cellular immunity, which is considered a promising therapeutic strategy in the immunotherapy of solid tumors (7). Currently, most cancer vaccines are still in the stage of preclinical and clinical research except for Sipuleucel-T (Provenge), a US FDA-approved vaccine for mCRPC (8). To sum up, these three main cancer immunotherapies are still associated with numerous challenges.

**Figure 1:**
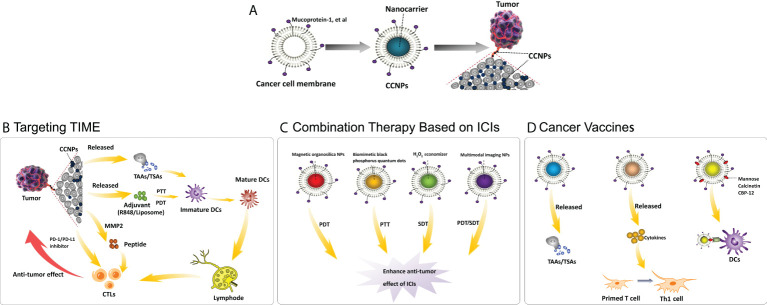
Application of cancer cell membrane-wrapped nanoparticles for cancer immunotherapy. **(A)** The structure of CCNPs; **(B)** Mechanism of CCNPs targeting on TME: CCNPs directly carry TSAs from cancer cell membranes or by different stimuli, such as PTT, then release TAAs. The released TSAs/TAAs, together with the immune adjuvant (R837/Liposome), drive the maturation of DCs. The CCNPs also used the tumor-specific enzyme metallomatrix protease 2 (MMP2) to dramatically extend the half-life of the peptides, e.g. PD-L1 inhibitory peptides, or combined with PD-1/PD-L1 inhibitors to further enhance the anti-tumor effect. The above processes promote the infiltration of CTLs for treating distant metastasis. **(C)** Combination therapy based on ICIs: A combination therapeutic strategy that includes PDT, PTT and SDT, particularly robust anti-cancer agents loaded on CCNPs, results in enhanced anti-tumor effect of PD-1/PD-L1 inhibitors. **(D)** Cancer vaccines: As tumor vaccines, CCNPs can directly release TSAs/TAAs, release cytokines to promote the transformation of functional immune cells or are added special molecules on the cancer cell membrane to increase the antigen presentation to DCs, thus further enhancing the anti-tumor immune effect. Abbreviations: CCNPs = cancer cell membrane-wrapped nanoparticles, TIME = tumor immune microenvironment, TAAs = tumor-associated antigens, TSAs = tumor-specific antigens, PTT = photothermal therapy, PDT = photodynamic therapy, MMP2 = metallomatrix protease 2, PD-L1/PD-1 = programmed death-ligand 1/programmed death-1, CTLs = cytotoxic T lymphocytes, DCs = dendritic cells, ICIs = immune checkpoint inhibitors, NPs = nanoparticles, SDT = sonodynamic therapy, PDT = photodynamic therapy, Th1 cell = T helper 1 cell, CBP-12 = 12-mer Clec9a binding peptide.

In the pursuit of achieving the broader goal of “curing cancer,” apart from determining the dominant drivers of cancer immunity, optimizing long-term survival with multi-agent cancer immunotherapy combination regimens is an important line of research (9). With regard to the second goal, the development of “smart-designed” nanoparticles (NPs) opens up the possibility of therapeutic advancement. NPs can be classified as organic and inorganic. Polylactic glycolic acid (PLGA) and indocyanine green are the most commonly used organic NPs and the only near-infrared dyes approved by the US FDA, respectively (10). Liposomes and gelatin are other organic NPs, which also have the advantages of biocompatibility, biodegradability and non-toxicity (11). On the other hand, mesoporous silica (the US FDA-approved), Fe_3_O_4_, gold, upconversion NPs, etc., are commonly used inorganic NPs (12). The metal tunable features and electrical, optical, thermal and magnetic properties of inorganic NPs bring a rich combination to the design (13). “Smart-designed” NPs could be used for the delivery of single or multiple therapeutic cargos and could be integrated with photodynamic therapy (PDT), photothermal therapy (PTT) and acoustic dynamic therapy (SDT) to further increase the effects of drugs (14–16). However, NPs are exogenous substances that are recognized by the immune system and by renal and hepatic clearance. Biomimetic nanoengineering of NPs wrapped with cell membranes can make up for this drawback. Unlike most other membrane donor cells, cancer cells are easy to culture in large volumes *in vitro* for mass membrane collection, and more importantly, cancer cell membranes (CCMs) inherit the functions of homologous targeting and antigen library from source cells and have been used in cancer-targeted therapy and cellular immunotherapy (15, 17–22). Hence, CCMs are the ideal candidate for the engineering of NPs for cancer therapy (23). The first study on these membranes showed that the MDA-MB-435 tumor cell membrane coating enabled approximately 40-fold and 20-fold increases in uptake compared with red cell NPs and bare PLGA cores, respectively (22). Since this initial work, the use of CCM-wrapped NPs (CCNPs) has become an emerging topic in the field of anti-tumor therapy and immunotherapy today. In the following sections, we present an exploration of the application of CCNPs in diverse immunotherapy approaches and combination therapy. We also offer a constructive view on the challenges involved in transferring the progresses in this technology from the laboratory to the clinic (**Figure 1**).

## Applications of membrane-wrapped nanoparticles in cancer immunotherapy

### Targeting or modulating the tumor immune microenvironment

The successful induction of anti-tumor adaptive immune response requires three elements: an antigen, an adjuvant, and a conducive immune microenvironment (24). To a great extent, the efficacy of cancer immunotherapy depends on the TME, especially the tumor immune microenvironment (TIME) (25). In CCMs, a wide range of molecules are retained on the surface of the source cancer cell, such as mucoprotein-1, epithelial adhesion moieties, lymphocyte-homing receptors, galectin-3, integrins, and cadherins, that could help CCNPs to escape the immune system (26–29). The use of CCMs introduces various characteristics of cancer cells to the nanocarriers, thus allowing the targeting of homotypic tumors and the development of personalized immunotherapy (30, 31). For example, Jin et al. demonstrated that MDA-MB-231-PLGA NPs disrupted the migration of cancer cells towards fibroblasts and increased the population of cytotoxic T lymphocytes (CTLs) in immune-competent mice (20). Further, Wu et al. showed that Fe_3_O_4_@SiO_2_ magnetic NPs bearing tumor-specific antigens (TSAs) on the surface effectively stimulated natural killer cells by enhancing the expression of surface-activating receptors and boosting anti-tumor function through the secretion of soluble cytotoxic effectors (32).

Combining CCNPs with adjuvant and/or tumor antigen technologies is a promising strategy to induce potent anti-tumor responses. Kim et al. developed a lipocomplex (4T1-liposome adjuvant-KillerRed) that has photocytotoxic and immunomodulatory properties, including dendritic cell (DC) maturation and production of CTLs following effective PDT (33). Similarly, Wang et al. reported an integrative photodynamic immunotherapy approach in which reactive oxygen species (ROS) triggered an anti-tumor immune cascade (34). In this approach, ovalbumin antigen was nano-packaged by establishing an intermolecular disulfide bond network between the antigens, which were employed as photosensitizer nanocarriers and subsequently coated with B16-OVA-CCMs (34).

CCNPs loaded with multiple drugs, such as chemotherapy drugs and ICIs, will have synergistic effects when used in combination therapy. For example, Cheng et al. showed that the intelligent biomimetic nanoplatform AM@DLMSN@CuS/R848 has a strong homogeneous targeting ability to mediate triple-negative breast cancer (TNBC)-targeted delivery of the immune adjuvant R848 and the PD-1/PD-L1 inhibitor AUNP-12 (35). In addition, AM@DLMSN@CuS/R848 could possess high photothermal efficiency that can ablate the primary tumors of TNBC and trigger the rapid release of R848 to produce vaccine-like functions against TNBC recurrence and metastasis (35). In another such study, Chen et al. reported a cocktail therapy based on “nano-targeted cells” (36). In this therapy, PTT was triggered by laser irradiation and helped the release of tumor-associated antigens (TAAs), thereby enhancing docetaxel-mediated immune effects (36). The released TAAs, together with the immune adjuvant R837, drove the maturation of DCs and secretion of cytokines, including TNF-α, IL-6, and IL-12 (36). Furthermore, docetaxel polarized protumoral M2-phenotype tumor-associated macrophages (TAMs) to tumoricidal M1-phenotype TAMs; this alleviated immunosuppression in the TME and was accompanied by a decrease in IL-10 (36). The above processes promoted the infiltration of CTLs for the treatment of distant metastasis (36). Fang et al. developed a multifunctional 2D ultrathin FePSe_3_@APP@CCM nanosystem with multimodal imaging ability, photothermal features, and PD-1-inhibiting capability (37). In terms of cancer immunotherapy, the nanosystem could trigger DC maturation and subsequent cytokine secretion to activate T cell-related immune responses following PTT (37). At the same time, the nanosystem could achieve immunotherapy with an anti-PD-1 checkpoint inhibitor strategy by blocking the PD-1/PD-L1 pathway (37). Wu et al. reported that biomimetic S-CM-HPAD NPs could not only modulate tumor immune escape by inhibiting myeloid-derived suppressor cell recruitment in tumors but also alter the TME by improving T-cell proliferation and stimulating the secretion of cytokines (38).

The low immunogenicity and TIME of tumors are negative for initiating and forwarding immune cycle, respectively (39, 40). Meng et al. developed an effective delivery system for peptides that target the TIME (41). The system used the tumor-specific enzyme metallomatrix protease 2 (MMP2) to dramatically extend the half-life of the peptides (60 times longer than the control group) (41). For example, prolongation of the half-life of the PD-L1 inhibitory peptide could maintain the reactivation capacity of T cells and inhibit tumor growth under both *in vitro* and *in vivo* conditions (41). In a similar strategy, Alsaiari et al. loaded nivolumab in biomimetic metal-organic frameworks (zeolitic imidazolate frameworks or ZIFs) that are known to enrich nivolumab within TIME, enhance the sensitivity of TIME to anti–PD-1 immunotherapy, and systemically activate a specific anti-tumor immune response enabled by the local inhibition of the regulatory T-cells (42). In a noteworthy innovative contribution by Yang et al., they proposed a potential anti-tumor strategy in which a smart biomimetic nanoplatform targets both tumor metabolism and immunity based on ROS-ferroptosis-glycolysis regulation (43).

Hybrid membranes can combine the properties of various cell membranes and have optimized function (44). Hao et al. developed chemoimmunotherapy delivery vehicles based on C6 cell membranes and DC membranes (DCm) to create hybrid membrane-coated docetaxel nanosuspensions (45). For cancer immunotherapy, this CNNP presents antigens to the immune system for efficient downstream immune activation based on the antigen presentation characteristic of DCm (46). Xiong et al. fused a murine-derived ID8 cancer cell-red blood cell hybrid membrane coated indocyanine green-loaded Fe_3_O_4_ magnetic NPs to treat ovarian cancer *via* synergistic photothermal-immunotherapy (46). In this study, the CCNP was characterized by a prolonged circulation lifetime in blood (46). This CCNP, in response to PTT, could induce tumor necrosis and release whole-cell tumor antigens, activate CD8^+^ cytotoxic T cells and reduce regulatory Foxp3^+^ T cells, and further enhance antitumor immunotherapy (46).

### Combination therapy with immune checkpoint inhibitors, photothermal and sono-photodynamic technologies

In recent years, immunotherapy with ICIs (commonly used anti-CTLA-4 antibodies in the clinic such as ipilimumab; anti-PD-1 antibodies such as nivolumab, pembrolizumab, camrelizumab, and sintilimab; and anti-PD-L1 antibodies such as atezolizumab and durvalumab) has shown promise for long-term survival in a fraction of patients with advanced or metastatic cancer, but off-target effects and drug resistance remain inevitable (47, 48). The reasons for the failure of ICI treatment include a lack of TAAs on the surface of tumor cells that can be recognized by immune cells, a lack of tumor-infiltrating lymphocytes (TILs), an immunosuppressive TME, and the presence of other suppressive immune checkpoints and suppressive cytokines (49, 50). These regimens combining ICIs with other technologies can eliminate negative regulators, overcome the immunosuppressive TME, increase T-cell survival, drive T-cell memory, and activate new immunity in patients without an existing strong immune response (9). Therefore, a combination therapeutic strategy that includes chemotherapy, PDT, PTT, SDT, and others, particularly robust anti-cancer agents loaded on CCNPs, may have immense potential.

As early as 2019, Xie et al. designed the CCNP method, in which mesoporous silica NPs (MSNs) are loaded with glucose oxidase and then wrapped with B16-F10 CCMs to realize starvation therapy together with anti-PD-1 antibody therapy and improved anti-cancer effects (51). In the same year, Wang et al. developed bullet-like Janus magnetic mesoporous organosilica NPs loaded with chlorine e6 and realized the combined application of PDT mediated with immunogenic NPs and magnetic hyperthermia to synergically enhance the anti-metastatic efficacy of anti-CTLA-4 antibody immunotherapy (52). Subsequently, Shao et al. and Zhao et al. reported that the combined treatment strategy of “doxorubicin-loaded or dacarbazine-loaded MONs coated with CCMs and anti-PD-1/PD-L1 antibody” has significant potential as a superior chemo-immunotherapy method (53, 54). Zhao’s team also developed a combined photothermal immunotherapy strategy based on CCM-encapsulated biomimetic black phosphorus quantum dots for tumor-targeted PTT and anti-PD-L1 antibody immunotherapy (55). Apart from PTT and hyperthermia, SDT is another promising site-specific tumor cell killing strategy that has the characteristics of being non-invasive and having high tissue penetration depth (56). Jiang et al. showed that the H_2_O_2_ economizer notably alleviates hypoxia-associated limitations in TME and that on-demand oxygen production-assisted sonodynamic immunotherapy can significantly inhibit 4T1 tumors (57). However, due to the complex and dynamic features of cancer occurrence and development, treatment with just one or two strategies may not be sufficient to meet the clinical needs (58). Therefore, combined anti-tumor therapy strategies exploring different therapeutic approaches and related mechanisms of action have broad clinical applications (59). It is worth noting that the serious side effects of combination therapy should not be ignored. Lin et al. designed and constructed a novel multimodal imaging nanoprobe fused with CNNPs loaded with superparamagnetic iron oxide and hematoporphyrin monomethylether, which augmented triple therapy with PTT, SDT, and ICI under the precise guidance of magnetic resonance, photoacoustic imaging, and photothermal imaging (60). In a recent innovative research, the use of cascade cell membrane (CCM and DCm) camouflaging to prepare biomimetic NPs was found to resolve the cross-priming of T cells, and it was applied in immunotherapy with a clinical anti-PD-1 antibody to induce powerful antigen-specific anti-tumor immunity in multiple mouse models of tumors, including B16-OVA, TC-1, and Hepa 1–6 tumors (61).

### Application in cancer vaccine therapy

Cancer vaccine therapy, together with ICI therapy and adoptive cellular therapy, are currently three of the most widely used cancer immunotherapy approaches (62–64). Sipuleucel-T (Provenge), a US FDA-approved vaccine for mCRPC in 2010, is the first therapeutic vaccine to be approved for any cancer (65). Cancer vaccines, especially cancer nanovaccines, have demonstrated effective cancer immunotherapy effects by active immune amplification of the tumor-specific T-cell response through the targeted and coded delivery of an antigen and adjuvant (66, 67). CCMs containing TAAs or TSAs have been widely used in the manufacture of cancer vaccines. To a large extent, the camouflage strategy with vaccines can ensure the targeting and effectiveness of tumor antigen presentation. Fontana et al. developed novel porous silicon­based nanovaccines that could enhance the secretion of IFN­γ in peripheral blood mononuclear cells but did not induce the secretion of IL­4; this oriented the polarization of the newly primed T­cells toward a Th1 cell­mediated response (68). Likewise, Cheng et al. produced a cancer vaccine by reconstructing the CCM and using monophosphoryl lipid A as a Toll-like receptor 4 agonist along with egg phosphatidylcholine; this vaccine could significantly enhance immune response and establish immune memory against a 4T1 challenge (69). The above two studies have provided promising candidates for the clinical translation of cancer vaccines.

As combination therapy is known to synergistically enhance anti-tumor effects, several researchers have also explored combination therapy with cancer vaccine. For example, Ye et al. integrated CCM-coated Black phosphorus quantum dots (BPQDs), granulocyte-macrophage colony-stimulating factor (GM-CSF), and lipopolysaccharide into a thermosensitive hydrogel that was subcutaneously injected for sustained delivery of the vaccine; this strategy as found to dramatically improve the survival rate of 4T1 or B16F10 tumor-bearing mice after surgery when it was combined with anti-PD-1 antibody immunotherapy and near-infrared irradiation (70). In terms of combined chemotherapy and PDT, Ni et al. explored a vaccine (a CCM coated with calcium carbonate NPs loaded with low-dose doxorubicin hydrochloride and chlorins e6) that could elicit TAAs and DC recruitment and trigger a subsequent immune response cascade (71). To further improve the efficacy of such nanovaccines, another promising strategy is to modify the surface of such CCNPs to afford them the ability to target DCs. Due to the specific binding of mannose and its receptors on DCs, mannose modification could promote the binding and cellular uptake of NPs by DCs and, consequently, enhance lymph node retention of the cancer vaccine for more effective *in vivo* vaccination (72). Calcinetin, as a representative of damage-associated molecular patterns, is expressed on the surface of the cancer vaccine; this could induce active uptake by DCs while activating immune memory cells to provide long-term protection (73). One study has shown that a 12-mer Clec9a-binding peptide (CBP-12) that specifically targets Clec9a can enhance antigen uptake and CD8^+^ T cell responses even in the absence of adjuvants (74). In another such study, Gou et al. developed an engineered CBP-12-expressed CCM-coated cancer vaccine to deliver a stimulator of interferon gene agonists and tumor antigens to Clec9a^+^ DCs (73). This cancer vaccine exhibited robust anti-tumor effects in both anti-PD-1-responsive and anti-PD-1-resistant tumor models, and when combined with radiotherapy, it had significant synergistic anti-tumor effects (75).

A much simpler and more interesting approach would be to directly modify the atypical design of immune adjuvants and/or aptamers to coat them on CCMs, rather than loading them inside the NPs and then coating those NPs with CCMs. Accordingly, Liu et al. designed a relatively simple strategy for equipping CCMs with functional DNA (including a CpG oligonucleotide, a Toll-like receptor 9 agonist as an immune adjuvant, and a DC-specific intercellular adhesion molecule-3-grabbing nonintegrin-targeted aptamer) as a targeted cancer vaccine that could induce powerful anti-tumor immune responses (76). Further, when this vaccine is combined with anti-PD-1 antibody immunotherapy, it could achieve the optimal therapeutic outcome in combating existing tumors and preventing tumor recurrence *via* an immune memory effect (76).

## Challenges and perspectives

With the rapid development of nanotechnology, synthetic nanocarriers play an increasingly important role in cancer immunotherapy. Stimuli-responsive nanocarriers enable the precise control of drug release through exposure to specific stimuli and exhibit excellent specificity in response to various stimuli. In particular, stimuli-responsive nanocarriers have evolved rapidly from single stimuli-responsive systems to multistimuli-responsive systems (77). However, one of the key challenges is that the size of most nanomaterials ranges from tens to hundreds of nanometers due to the limitations of the preparation methods. Nanocarriers with large size (≥200 nm) are primarily accumulated in extracellular spaces while nanocarriers with small sizes (≤10 nm) can easily be filtered out through the kidneys (78). Even more crucial is that the immune system recognizes and eliminates the majority of nanocarriers as foreign substances. Hence these disadvantages make it difficult for nanocarriers to meet clinical standards. CCNPs possess beneficial characteristics, such as prolonged drug delivery, immunological evasion, homotypic targeting, longer blood circulation, and specific ligand/receptor recognition, which can overcome major barriers in cancer immunotherapy (79).

In the future, the emphasis should be on the recovery of specific immunosuppressive pathways in anti-tumor processes, rather than merely boosting the broad and untargeted systemic immune response (1). Specifically, the following research pathways would be beneficial: determining whether TIME is caused by the tumor, focusing on immunosuppression of the TME, and identifying new targets in the major functional pathways (1). Existing experimental CCNPs can simulate part of the TME, but cannot fully adapt to the complex and changeable TIME, especially in the human body. This is one of the key reasons for primary and secondary drug resistance in various types of immunotherapy and even in immunotherapy combination regimens. Hybrid membrane nanocarriers may have some advantages because they can combine the properties of a variety of cell membranes (e.g., RBCs, WBCs, and platelets including DCs and T cells) and optimize their function. Of particular concern, however, is that more complex CCNPs imply greater immunogenicity and toxicity, which may cause unwanted immune responses. In addition, the concern about injecting cancer cell-derived substances into the body is valid, and healthy people may be reluctant to accept this technology as a preventive vaccine in particular. In addition to providing more medical education to the public, technical optimizations and rigorous procedures must be developed to ensure the purity of the membrane wrapping, that is, to ensure that it does not contain any molecules that may promote cancer growth. Another important dilemma to consider for clinical use is that this approach may be appropriate only for patients already diagnosed with cancers that have been surgically removed, as the required amount of cancer cells can be obtained from the resected tissue (80). Some other challenges in isolating primary cancer cells from biopsy samples are the procedures for eliminating the extracellular matrix and the conditions required to grow it in a way that prevents significant changes in the antigen profiles (81, 82). In addition, the need for large-scale cell culture and industrialization are also key challenges that may require further breakthroughs in biomedical technology.

In due course, to translate this technology to the clinic, the following points may be urgently needed breakthroughs. First of all, the complexity of various synthetic nanocarriers hinder their large-scale uses, we should always consider simplifying in the design and then screening several nanocarriers with more promising clinical application and apply them to clinical trials to verify their effectiveness and safety. Secondly, the powerful advantage of “mimicking nature” is that cell membrane materials have the advantage of biologically active vesicles. A similar and standardized manufacturing process will be required in order to develop standardized or quantified bioactive membrane-coated nanocarriers. Finally, aside from the technical difficulties, the concept of prioritizing treatment for cancer patients in well-designed clinical trials should be widely understood and practiced throughout society. In the future of translational medicine, we hope that it will be possible to obtain CCMs from primary or metastatic tumor lesions and develop personalized CCNPs for cancer immunotherapy.

## Conclusions

This review has highlighted the current major developments in CCNPs for cancer immunotherapy. CCNPs have enormous potential as powerful vehicles to enhance cancer therapy, but there are immense challenges in translating their effects *in vitro* to the clinical setting. The future of this field lies in solving this issue and providing tailormade, personalized treatment to individual patients based on CCNPs and CCNPs combined with other effective therapies. While this objective is challenge, it will continue to drive research around CCNPs.

## Author contributions

PS and JR contributed to the conception and design this study. QJ, RC, FY, CY, QL, SX and WW conducted the initial literature review and selected the studies for inclusion. QJ and MX wrote the initial draft of the manuscript. YJ, PS, and JR revised the draft and approved the final submission of the study. All authors contributed to the article and approved the submitted version.

## Funding

This work was supported by fund from the National Natural Science Foundation of China (No.82100161 and No.81874173) and the Natural Science Foundation of Zhejiang Province (No. LQ22H080004 and No. LY22H160019).

## Conflict of interest

The authors declare that the research was conducted in the absence of any commercial or financial relationships that could be construed as a potential conflict of interest.

## Publisher’s note

All claims expressed in this article are solely those of the authors and do not necessarily represent those of their affiliated organizations, or those of the publisher, the editors and the reviewers. Any product that may be evaluated in this article, or claim that may be made by its manufacturer, is not guaranteed or endorsed by the publisher.

## Abbreviations

CAR-T, chimeric antigen receptor T-cell immunotherapy; TME, tumor microenvironment; TGF, transforming growth factor; mCRPC, metastatic castration-resistant prostate cancer; ICIs, immune checkpoint inhibitors; PD-L1/PD-1, programmed death-ligand 1/programmed death-1; CTLA-4, cytotoxic T-lymphocyte-associated protein 4; NPs, nanoparticles; PLGA, polylactic glycolic acid; PDT, photodynamic therapy; PTT, photothermal therapy; SDT, sonodynamic therapy; CCMs, cancer cell membranes; CCNPs, cancer cell membrane-wrapped nanoparticles; TIME, tumor immune microenvironment; CTLs, cytotoxic T lymphocytes; TSAs, tumor-specific antigens; DC, dendritic cell; ROS, reactive oxygen species; TNBC, triple-negative breast cancer; TAAs, tumor-associated antigens; TAMs, tumor-associated macrophages; MMP2, metallomatrix protease 2; DCm, dendritic cell membrane; TILs, tumor-infiltrating lymphocytes; MSNs, mesoporous silica NPs; BPQDs, Black phosphorus quantum dots; GM-CSF, granulocyte macrophage colony-stimulating factor; CBP-12, 12-mer Clec9a-binding peptide.
